# B7-H1-Deficiency Enhances the Potential of Tolerogenic Dendritic Cells by Activating CD1d-Restricted Type II NKT Cells

**DOI:** 10.1371/journal.pone.0010800

**Published:** 2010-05-24

**Authors:** Carolin Brandl, Sonja Ortler, Thomas Herrmann, Susanna Cardell, Manfred B. Lutz, Heinz Wiendl

**Affiliations:** 1 Institute of Virology and Immunobiology, University of Wuerzburg, Wuerzburg, Germany; 2 Department of Neurology, University of Wuerzburg, Wuerzburg, Germany; 3 Department of Microbiology and Immunology, Institute of Biomedicine, Goteborg University, Goteborg, Sweden; New York University, United States of America

## Abstract

**Background:**

Dendritic cells (DC) can act tolerogenic at a semi-mature stage by induction of protective CD4^+^ T cell and NKT cell responses.

**Methodology/Principal Findings:**

Here we studied the role of the co-inhibitory molecule B7-H1 (PD-L1, CD274) on semi-mature DC that were generated from bone marrow (BM) cells of B7-H1^−/−^ mice and applied to the model of Experimental Autoimmune Encephalomyelitis (EAE). Injections of B7-H1-deficient DC showed increased EAE protection as compared to wild type (WT)-DC. Injections of B7-H1^−/−^ TNF-DC induced higher release of peptide-specific IL-10 and IL-13 after restimulation *in vitro* together with elevated serum cytokines IL-4 and IL-13 produced by NKT cells, and reduced IL-17 and IFN-γ production in the CNS. Experiments in CD1d^−/−^ and Jα281^−/−^ mice as well as with type I and II NKT cell lines indicated that only type II NKT cells but not type I NKT cells (invariant NKT cells) could be stimulated by an endogenous CD1d-ligand on DC and were responsible for the increased serum cytokine production in the absence of B7-H1.

**Conclusions/Significance:**

Together, our data indicate that BM-DC express an endogenous CD1d ligand and B7-H1 to ihibit type II but not type I NKT cells. In the absence of B7-H1 on these DC their tolerogenic potential to stimulate tolerogenic CD4^+^ and NKT cell responses is enhanced.

## Introduction

DC are not only potent inducers of adaptive immune responses but also mediators of tolerance. Immature DC, which do not express maturation markers and produce no cytokines are considered to be tolerogenic. Fully mature DC upregulate maturation markers upon stimulation and produce cytokines and are therefore termed immunogenic. In addition another maturation stage has been characterized as semi-mature DC [1]. These cells which upregulate maturation markers but do not produce cytokines are tolerogenic and can protect mice from EAE by inducing protective CD4^+^ T cell and NKT cells responses [2], [3].

For efficient T cell activation and the generation of functionally competent effector cells, additional costimulatory signaling through surface molecules on APC and T cells is needed [4]. Upregulation of T cell activity by attenuation of coinhibitory signals provides an attractive tool for the treatment of autoimmune and inflammatory diseases [5]. Among the B7-CD28 family of costimulatory molecules, B7-H1 on APC has been proposed to regulate immune responses by interaction with PD-1 or CD80 (B7-1) and possibly another yet unidentified receptor on activated T cells [6], [7], [8]. B7-H1 is widely expressed on different cell populations including T and B cells, monocytes, DC, but also non-hematopoietic cells [9], [10]. Its receptor PD-1 is inducible on T and B cells upon activation [11]. Due to their broad tissue expression pattern, B7-H1/PD-1 interactions have been implicated in critically modulating parenchymal inflammation, limiting autoimmune responses and confining immune cell functions in the periphery [12], [13], [14], [15].

Originally, interactions of B7-H1/PD-1 were postulated to costimulate T cell activation and proliferation [16], but more recent data point towards a negative regulatory role of the B7-H1/PD-1 pathway in T cell activation, proliferation and CTL activity [6], [9], [17], [18]. Using the animal model of MS, EAE, this coinhibitory effect mediated by B7-H1 has been extensively studied [12], [15], [19], [20], [21], [22]. It was demonstrated that systemic absence of B7-H1 renders 129Sv mice susceptible to EAE and exacerbates disease in C57BL/6 mice [6], [15], [21]. Additionally, blockade of B7-H1 or PD-1 with specific antibodies resulted in increased susceptibility and progression of EAE in various mouse strains [20], [23]. While B7-H1 expression in the CNS was shown to be very limited under physiological conditions, it is rapidly upregulated during inflammation [19]. Particularly microglial cells expressing inhibitory B7-H1 were found to dampen encephalitogenic T cell responses in later stages of EAE, suggesting a major contribution of this molecule to confinement of parenchymal inflammation [12], [22]. Similar conclusions were drawn from experiments using an animal model of diabetes, where PD-1/B7-H1 interactions were shown to regulate effector cell differentiation of autoreactive CD8^+^ T cells during the presentation of tissue antigens [13]. However, little is known about the role of the B7-H1/PD-1 pathway in mediating tolerogenicity during protection against neuroinflammation.

In this study, we investigated the role of B7-H1 on tolerogenic DC in mediating tolerance induction in the context of autoimmune inflammation. After intravenous injections of TNF- treated semi-mature DC deficient in B7-H1 we found a stronger tolerogenic capacity in EAE protection in comparison to WT DC. In the CNS, we found no differences in total CD4 and CD8 T cell infiltration, but lower numbers of neuroantigen-specific IFN-γ and IL-17 producing cells. The ameliorated EAE phenotype was accompanied by increased production of the protective cytokines IL-10 and IL-13 and reduced levels of proinflammatory IFN-γ and IL-17 in the periphery. Additionally, we observed higher serum cytokine levels of IL-4 and IL-13 in B7-H1^−/−^ DC injected mice, which contributed to the tolerance induction. We found that type II cells but not type I invariant NKT (iNKT) cells were the producers of these cytokines. It has been described that the B7-H1/PD1 pathway is involved in the activation of iNKT cells [24], [25]. We found that the signaling via B7-H1 on the DC does not influence the iNKT response but it has an impact on other CD1d-restricted cells. It has been shown that in mice lacking the MHC class II molecule there is still a population of T cells other than iNKT cells, which recognize the CD1d molecule. Now we show that these type II NKT cells are regulated by B7-H1 [26], [27]. Our data describe the importance of B7-H1/type II NKT cell interactions and its unexpected impact by modulation of tolerogenic DC in EAE protection.

## Results

### DC from WT and B7-H1^−/−^ mice show a similar expression of surface markers and have the same potential to stimulate CD4^+^ T cells

**Figure 1:**
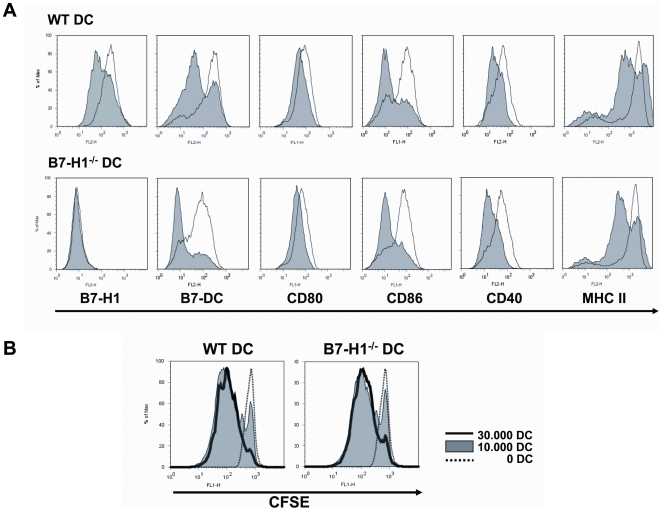
WT and B7-H1^−/−^ DC show equal expression of surface markers and have the same potential to stimulate CD4^+^ T cells. A) BM-derived DC from WT and B7-H1^−/−^ mice were matured with TNF over night, stained for different surface markers and analyzed by flow cytometry. The shaded histograms show the unstimulated control and the black lines show TNF-matured DC. Results are representative for 3 independent experiments. B) TNF-stimulated and MOG_35–55_-loaded DC from WT and B7-H1^−/−^ mice were co-cultured with CFSE-stained CD4^+^ T cells with MOG_35–55_ transgenic TCR. Proliferation of the T cells was analysed by the dilution of the CFSE-staining. The dottet line shows the control without DC, the shaded histogram shows the co-culture with 1×10^4^ DC and the filled line shows the co-culture with 3×10^4^ DC. Results are representative for 3 indepentent experiments.

We compared TNF-matured DC from WT and B7-H1^−/−^ mice regarding their surface markers and their stimulatory capacity. FACS analysis of these DC showed a comparable expression of CD80, CD86, CD40, MHC II and B7-DC (PD-L2) on their cell surface and despite the loss of B7-H1 in the knock-out mice (Figure 1A). We further investigated the potential of WT and B7-H1^−/−^ DC to stimulate T cells. Therefore enriched CD4^+^ T cells derived from 2D2 mice bearing a TCR specific for MOG_35–55_ were co-cultured with MOG-loaded DC from WT and B7-H1^−/−^ mice. The CD4^+^ T cells show comparable proliferation rates after incubation with WT or B7-H1^−/−^ DC as indicated by CFSE-dilution (Figure 1B).

### Higher tolerogenic potential of B7-H1^−/−^ DC in EAE

**Figure 2:**
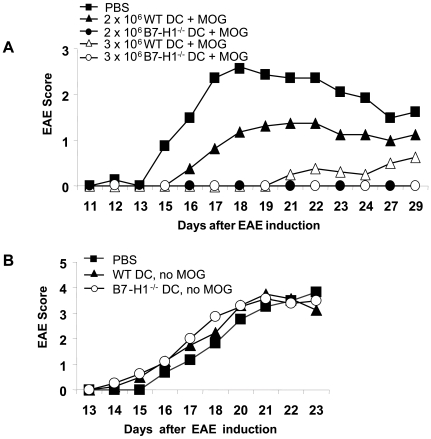
B7-H1^−/−^ DC have a higher tolerogenic potential than WT DC. A) 2×10^6^ or 3×10^6^ MOG_35–55_ loaded and TNF-matured WT or B7-H1^−/−^ DC were injected i.v. into WT mice 7, 5 and 3 days before EAE induction. Control mice were injected with PBS. On day 0, EAE was induced and disease course was monitored daily. Results represent the average disease score of 4 mice per group and are representative for 4 independent experiments. B) 2,5×10^6^ TNF-matured, but not MOG_35–55_ loaded WT or B7-H1^−/−^ DC were injected i.v. into WT mice 7, 5 and 3 days before EAE induction. Results represent the average disease score of 4 mice per group and are representative for 2 independent experiments.

We previously demonstrated that repetitive i.v. injections of semi-mature DC, which were stimulated with TNF and loaded with MOG_35–55_ peptide, were able to protect mice from developing EAE [3]. Mechanisms involved in the mediation of tolerance revealed the induction of IL-10 producing CD4^+^ T cells as well as the activation of NKT cells, which in turn rapidly produced protective cytokines contributing to the tolerogenic capacity of the semi-mature DC [2], [3]. To investigate the role of the coinhibitory molecule B7-H1 on tolerance induction we injected WT mice 7, 5 and 3 days before EAE induction with different numbers of MOG_35–55_ peptide loaded and TNF-matured WT and B7-H1^−/−^ DC. We found that PBS-injected control mice developed severe EAE, whereas TNF-DC-injected mice were partially or fully protected from disease depending on the number and phenotype of applied TNF-DC (Figure 2A, Table 1). Mice injected with a suboptimal dose of WT TNF-DC (2×10^6^) displayed a slightly ameliorated EAE course compared to control animals, increase in TNF-DC numbers resulted in a more pronounced protective phenotype. In contrast, B7-H1^−/−^ TNF-DC injected mice were fully protected from clinical signs of EAE, even at suboptimal doses of TNF-DC. To assess antigen specificity requirements for EAE tolerance induction, mice were treated with TNF-matured DC but without MOG_35–55_ before EAE induction. Here, no protective effect of both WT and B7-H1-deficient DC was observed, indicating that the induction of tolerance by injection of DC is neuroantigen-specific and dependent on the presentation of MOG_35–55_ peptide by DC (Figure 2B).

**Table 1:** Statistics of EAE.

Treatment	Incidence	Mean Day of Onset	Mean Maximal Score
PBS	16/16	13.9 (+/−2.1)	3.2 (+/−0.9)
WT DC	17/21	15.4 (+/−4.7)	1.7 (+/−1.1)^a^
B7-H1^−/−^ DC	10/20	15.0 (+/−3.7)	0.9 (+/−1.2)^a^^,^ b

Mice were injected i.v. (days −7, −5, −3) with PBS, WT DC or B7-H1^−/−^ DC and on day 0 EAE was induced. The incidence, mean maximal score and mean day of onset is shown.

a Statistically significant when compared with PBS group: p<0.001 by Student's t-test.

b Statistically significant when compared with WT DC group: p<0.05 by Student's t-test.

### Injection of B7-H1−/− TNF-DC reduces the frequency of neuroantigen-specific cytokine-secreting T cells in the CNS

**Figure 3:**
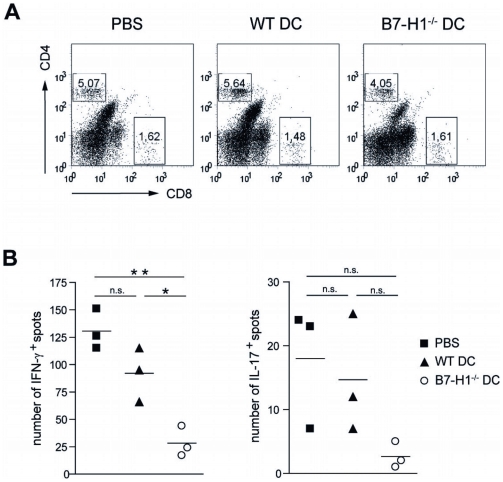
Injection of B7-H1^−/−^ DC results in reduced frequency of neurantigen-specific IFN-γ and IL-17 secreting cells in the CNS. MOG_35–55_ loaded and TNF-matured WT or B7-H1^−/−^ DC (2,5×10^6^) were injected i.v. into WT mice 7, 5 and 3 days before EAE induction. Control mice were injected with PBS. After 15 days CNS mononuclear cells were harvested and CNS T cell infitration and cytokine production were investigated using flow cytometry and ELISPOT assay. A) Dot blots are gated for live lymphocytes and inserted numbers represent the percentage of CD4^+^ and CD8^+^ CNS infiltrating T cells of 3–5 pooled mice per group of one experiment. Results are representative for 2 independent experiments. B) ELISPOT analysis of IFN-γ (left) or IL-17 (right) production by infiltrating CNS cells after MOG_35–55_ peptide restimulation. Results are representative for 2 independent experiments with 3–5 pooled mice per group. * p<0.05, ** p<0.01, n.s. not significant.

To address the amount of total T cell infiltration in the target organ of EAE-affected mice, we performed flow cytometry to quantify the abundance of CNS infiltrating T cells in the inflamed brains and spinal cords. 15 days after EAE induction an equal percentage of both CD4^+^ and CD8^+^ T cell populations could be detected in all three investigated groups (Figure 3A). To further characterize the cytokine production by CNS-infiltrating T cells, ELISPOT analyses following MOG_35–55_ restimulation revealed highest frequency of neuroantigen-specific IFN-γ and IL-17-producing cells in the CNS of PBS-injected mice (Figure 3B). WT TNF-DC injection resulted in a noticeable and consistent reduction of both IFN-γ (92±14 spots) and IL-17 (15±5 spots) producing CNS cells, although the differences were not statistically significant in comparison to the control group (130±11 spots for IFN-γ, 18±6 for IL-17) in two performed experiments. In contrast, using B7-H1-deficient TNF-DC injection prior to EAE induction, a marked decline in IFN-γ positive spots to 28±8 spots could be observed in the CNS of disease-protected animals. Again, the frequency of IL-17 secreting cells was reduced, but differences did not yield statistical significance (3±1 spots). CNS localized IL-10 production by MOG_35–55_ specific cells was also assessed but found undetectable in either group of mice (data not shown).

These data provide evidence that TNF-DC injection does not considerably influence total CNS T cell infiltration, but rather modulates the local cytokine milieu. Correlating to the EAE disease course, proinflammatory cytokines are substantially reduced upon B7-H1^−/−^ TNF-DC administration.

### Injection of B7-H1^−/−^ TNF-DC modulates primary neuroantigen-specific T cell responses in the periphery

**Figure 4:**
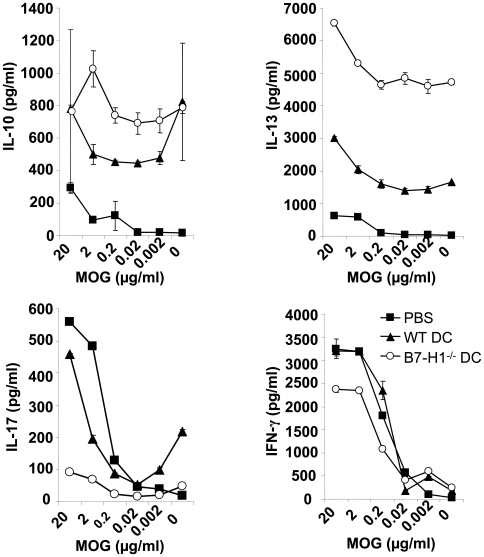
B7-H1^−/−^ DC induce more protective cytokines than WT DC. MOG_35–55_ loaded and TNF-matured WT or B7-H1^−/−^ DC (2,5×10^6^) were injected i.v. into WT mice 7, 5 and 3 days before EAE induction. Control mice were injected with PBS. 10 days after EAE induction spleen cells were isolated and restimulated with different concentrations of MOG_35–55_. After 4 days of culture supernatants were tested by ELISA for IL-10, IL-13, IL-17 and IFN-γ. Results show the mean of 4 mice per group and are representative for 2 independent experiments with 4 mice per group.

To assess the mechanism of increased EAE suppression in the absence of B7-H1 on tolerogenic DC, the spleens of PBS- or TNF-DC-injected mice were removed 10 days after EAE induction and peripheral T cell responses were investigated. Splenocytes were challenged with MOG_35–55_ peptide and after 72 hours cell culture supernatants were analyzed for their cytokine content by ELISA. In PBS-injected control mice the predominant cytokines that could be detected after MOG_35–55_ peptide titration were proinflammatory IFN-γ and IL-17, whereas only little protective IL-10 and IL-13 were present (Figure 4). By contrast, splenocytes from WT TNF-DC-injected mice produced large amounts of IL-10 and IL-13 even in the absence of or under low concentrations of neuroantigen. Simultaneously, secreted levels of IFN-γ were similar to the control group and IL-17 was modestly reduced compared to PBS-injected mice. Interestingly, this TNF-DC-mediated effect on cytokine profile modulation was even more pronounced in the absence of B7-H1 on injected TNF-DC. Here, we found a markedly reduced production of IFN-γ that was dependent on the MOG_35–55_ peptide concentration during spleen cell culture. Similarly, only small amounts of IL-17 could be detected in the supernatants. However, in B7-H1^−/−^ TNF-DC-injected mice peripheral levels of IL-13 were dramatically increased compared to both PBS- and WT TNF-DC-injected mice. This effect was found to be only partially neuroantigen-restricted, as higher concentrations of MOG_35–55_ peptide further enhanced IL-13 production. High peripheral IL-13 concentrations were also accompanied by an increased basal amount of IL-10 in B7-H1^−/−^ DC injected animals.

Together, these data indicate that TNF-DC injections induce the production of protective cytokines and downregulates potentially pathogenic master cytokines. In correlation with the reduced disease courses, this effect is more distinct in the absence of B7-H1 on TNF-DC.

### Injection of B7-H1^−/−^ TNF-DC induces increased production of protective serum cytokines, which are predominantly produced by type II but not type I NKT cells

**Figure 5:**
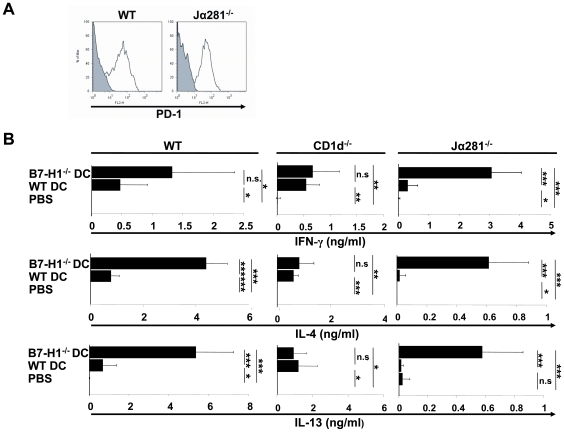
Strong increase of protective serum cytokines after injection of B7-H1^−/−^ DC injection is mainly produced by type II NKT cells. A) Splenocytes from WT and Jα281^−/−^ mice were stained for CD3 and NK1.1 and investigated by flow cytometry. Double positive cells (NKT cells) were analyzed for PD-1 expression. B) MOG_35–55_ loaded and TNF-matured WT or B7-H1^−/−^ DC (2,5×10^6^) were injected i.v. into WT, CD1d−/− or Jα281^−/−^ mice three times (on day −4, −2 and 0). Similarly, control mice were injected with PBS. Blood was collected 2 hours after the last DC injection and serum cytokines were measured by ELISA for IFN-γ, IL-17, IL-4 and IL-13. The results represent the average of 3 mice per group and are representative for 4 independent experiments for the WT mice and for 2 independent experiments for CD1d−/− and Jα281^−/−^ mice. * p<0.05, ** p<0.01, *** p<0.001, n.s. not significant.

We showed previously that TNF-DC induce a rapid production of type 2 cytokines detectable in mouse sera early after the third injection of TNF-DC and their contribution to EAE prevention by constraining Th1 and Th17 effector cell development [2]. As a prerequisite we tested the PD-1 expression on the cell surface by type I and II NKT cells of WT mice and the type II NKT cells of Jα281^−/−^ mice and could not find major differences (Figure 5A). To determine the relevance of TNF-DC B7-H1 expression for protective cytokine production, sera from WT or B7-H1^−/−^ TNF-DC-injected mice were collected and analyzed for cytokine profile by ELISA. While IL-17 concentration was generally low and remained unaffected by TNF-DC treatment (data not shown), TNF-DC-injected mice displayed significantly elevated levels of IFN-γ in their sera (Figure 5B). This effect was even reinforced in the absence of B7-H1 on TNF-DC. Simultaneously, IL-4 and IL-13 concentrations were markedly increased in WT TNF-DC-injected mice compared to control mice but significantly amplified using B7-H1-deficient TNF-DC. These findings are in line with the reduced disease course and support the notion of an increased induction of IL-4 and IL-13 producing cells in the absence of inhibitory B7-H1 on DC.

To identify the cell type responsible for increased IL-4 and IL-13 production upon transfer of TNF-DC, CD1d^−/−^ mice as well as Jα281^−/−^ were used as recipients of TNF-DC injections. CD1d^−/−^ mice were described previously to lack type I and type II NKT cells, whereas Jα281^−/−^ are deficient for the Vβ14-Jα18 NKT (type I) cell population [28], [29]. After three injections of WT or B7-H1^−/−^ TNF-DC in CD1d^−/−^ mice serum samples were taken and subjected to cytokine quantification by ELISA. Although Jα281^−/−^ mice have a bias towards high IFN-γ and lower IL-4 and IL-13 secretion as compared to the other mouse strains, we observed comparable relative shifts for the production of the cytokines IFN-γ, IL-4 and IL-13 in both TNF-DC-treated groups (Figure 5B). The absence of B7-H1 on TNF-DC did not lead to an augmented protective cytokine production in CD1d^−/−^ mice. These data suggest an involvement of type I or II NKT cells in cytokine release. To further dissect the implication of these two cell subsets, we addressed selectively the role of type I NKT cells by using Jα281^−/−^ mice. Serum ELISA revealed significantly increased IFN-γ, IL-4 and IL-13 concentrations in the B7-H1^−/−^ TNF-DC injected mice compared to WT TNF-DC-injected animals (Figure 5B). This indicates that type II NKT cells, but not type I NKT cells were the main producers of type 2 cytokines mediating protection from EAE.

Together, these data provide the first evidence of a direct inhibitory function of B7-H1 on TNF-DC for the induction of type II NKT cells and the loss of this negative signal is correlated with an amplified protective immune response.

### NKT cell lines express PD-1 but only type II NKT cells are stimulated by DC and are negatively regulated by B7-H1

**Figure 6:**
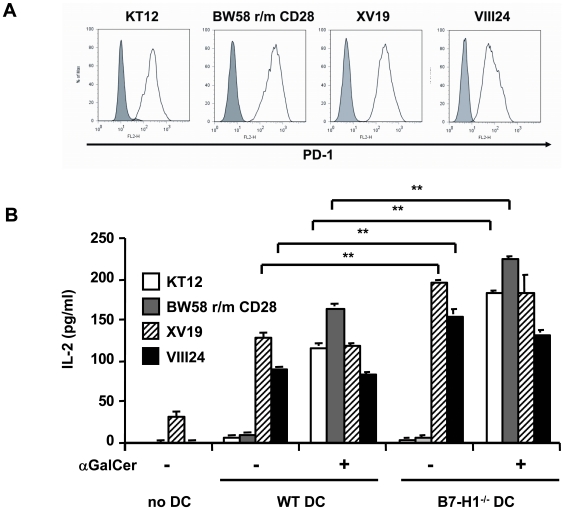
NKT cell hybridoma cells express PD-1 but only the type II NKT cells are stimulated by DC and are negatively regulated by B7-H1. A) NKT cell hybridoma cells s were stained for PD-1 and analyzed by FACS. The shaded histogram shows the isotype control staining and the black line shows PD-1 staining. B) DC from WT and B7-H1^−/−^ mice were co-cultured with the indicated NKT hybridoma cells in the presence or absence of αGC (10 ng/ml). After 24 hours, the supernatants were tested for IL-2 content by ELISA. Results show 1 out of 4 representative and independent experiments. ** p<0.01.

To further investigate if especially type II NKT cells were regulated by B7-H1 on DC we used different NKT cell lines, which were either related to type I (KT12 and BW58 r/m CD28) or type II NKT cells (XV19 and VIII24). All cell lines expressed PD-1, the ligand for B7-H1, at comparable levels (Figure 6A). We co-cultured DC from WT or B7-H1^−/−^ mice with the different cell lines and found that only the type II NKT cells responded to the DC, which was indicated by the production of IL-2. Type I-related cell lines did only respond to DC in the presence of the CD1d-ligand αGC. The IL-2 response of the type II-related cells was even higher in the absence of B7-H1 on the DC showing that B7-H1 negatively regulates these cell lines (Figure 6B). It is of note that also the type I NKT cell lines showed an increased IL-2 production in the presence of αGC by using DC that were generated from B7-H1^−/−^ mice (Figure 6B).

Taken together we could show that DC only activated type II NKT cells by their endogenously expressed CD1d-ligands. This interaction was regulated by B7-H1 on the surface of the DC and independent of the PD-1 expression by the NKT cell lines.

## Discussion

In this study we showed that absence of B7-H1 expression on semi-mature DC improves active tolerance induction upon DC injection into mice, which leads to the priming of protective peptide-restricted CD4^+^ T cells and CD1d-restricted type I and II NKT cells as well as the release of IL-10, IL-4 and IL-13. Injections of B7-H1^−/−^ DC induced higher amounts of protective cytokines, specifically from type II NKT cells as indicated by the results from CD1d^−/−^ and Jα281^−/−^ mice. Thus, our data point to an inhibitory role of DC-expressed B7-H1 for type II NKT cells and MHC II/peptide-restricted CD4^+^ T cells but not type I NKT cells. The NKT cell line experiments may indicate a lack of endogenous CD1d-ligands on the DC for presentation to type I NKT cells but not type II NKT cells.

Tolerance induction by TNF-DC is predominantly dependent on the presence of the MOG-peptide loading and thereby on MHC II/peptide-restricted CD4^+^ T cells, while CD1d-restricted NKT cells also contribute to the protection, however, by recognizing an endogenous CD1d-ligand from the DC that is irrelevant for the EAE [2]. Immediately after the third DC injection, CD4^+^ T cells and NKT cells released IL-4 and IL-13 to create an immune-deviatory environment that counteracts the Th1 and Th17 effector cells which mediate EAE. However, the use of CD1d-deficient DC did not allow a distinction between responses of type I or II NKT cells. Subsequently during the response, IL-10-producing CD4^+^ T cells dominate among the MOG-peptide specific populations in the spleens of mice that were completely protected from EAE [3]. Intracerebral injections of semi-mature DC were also able to delay or prevent EAE, as higher numbers of IL-10-producing neuroantigen-specific lymphocytes were generated in the periphery, thereby restricting IL-17 production in the CNS [30]. The surprising finding was that obviously “matured” DC could still act tolerogenic, which did not fit into the paradigm of DC maturation where immature DC were postulated to be tolerogenic and mature DC to be immunogenic [31]. This dilemma could be best circumvented by the proposal of a third maturation stage that was termed semi-mature [1]. These semi-mature DC expressed high levels of costimulatory molecules such as CD80, CD86 and CD40, which were required for the EAE-protection since immature DC were unable to protect. In contrast to LPS-treated and thereby fully mature DC, semi-mature DC did not release proinflammatory cytokines and were generally described as low cytokine producers [3]. The question remained whether the additional expression of coinhibitory molecules on semi-mature DC could contribute to the tolerogenic functions.

We and others previously demonstrated that APC-derived B7-H1 plays a critical negative regulatory role to T cell stimulation in the context of autoimmune CNS inflammation [6], [15], [20], [21], [30]. Using B7-H1^−/−^ mice in the MOG_35–55_ EAE model, earlier and exacerbated primary neuroantigen-specific immune responses were described ultimately leading to an aggravated disease course accompanied by higher immune cell infiltration in the CNS of B7-H1 deficient animals [21]. Thus, B7-H1 evolved as a crucial immune-inhibitory molecule capable of downregulating neuroantigen-specific T cell responses both in the periphery and the target organ of autoimmune inflammation. Recently, this concept was further corroborated by studies investigating the role of PD-1 ligands on different CNS APC applying a relapsing EAE model [22]. Therefore, we speculated that mediation of T cell tolerance induction might be altered in the absence of B7-H1 on APC [4], [11].

Surprisingly, we found here that in the absence of B7-H1 on the semi-mature DC their tolerogenicity was further enhanced. The enhanced release of protective cytokines could be attributed to the rare population of CD1d-restricted CD4^+^ T cells, so-called type II NKT cells. This is indicated by the use of specific type I and II NKT cell lines and the fact that the increased serum cytokine release of IL-4 and IL-13 after injection of B7-H1-deficient DC returned to the levels of mice injected with WT-DC when B7-H1-deficient DC were applied to CD1d^−/−^ mice which lack both type I and II NKT cells. In contrast, Jα281^−/−^ mice which lack only the Vα14-Jα18^+^ type I NKT cells but not the type II NKT cells [29], did show the serum cytokine increase. This is a strong indication that type II NKT cells are the predominant cell population that was affected by B7-H1 molecules on the DC. Type II NKT cells are selected in the thymus but on CD1d molecules instead of MHC II/peptide complexes. However, some authors found that they may not express markers typical for type I NKT cells such as NK1.1 [26], [27], [29] although this could not be confirmed in a type II NKT cell TCR transgenic mouse [32]. This may indicate the existence of NK1.1 negative and positive subtypes of type II NKT cells. A role for B7-H1 (PD-L1) interaction with PD-1 on type I NKT cells has recently been indicated to be responsible for the induction of an anergic state of type I NKT cells. This type I NKT cell anergy was defined as the fact that type I NKT cells become refractory for further stimulations by the ligand αGC after primary activation in vitro [24], [25]. The same anergic state is achieved after αGC injection in vivo [33]. However, we could not observe this when using CD1d-expressing DC for the type I and II NKT cell activation *in vivo* [2]. The discovery of sulfatide as an endogenous ligand for type II NKT cells that has been shown to influence EAE [34] and this may lead to anergy of type I NKT cells as shown in tumor and liver disease models [35], [36]. This opens the possibility that our DC may present sulfatide or a similar endogenous ligand to activate type II NKT cells leading to anergy or regulatory activity of type I NKT cells as discussed elsewhere [37].

Additionally, the fact that αGC primed NKT cells also do not polarize selectively into IL-4 or IFN-γ releasing cytokines *in vivo* but NKT cells do so when primed by differentially matured DC [2], [38], indicates that the priming conditions by αGC or DC may differentially affect type I and II NKT cell responses. This may be explained by the fact that that in our previous experimental settings [2], we did not distinguish between type I and II NKT cells. However here, by using the specific NKT cell lines it became clear that our in vitro generated DC do not carry an endogenous CD1d-ligand recognized type I NKT cell lines. In contrast, the same DC were readily detected by the type II NKT cell lines. However, type I NKT cells responded readily when pulsed with exogenous αGC. The differential capacities of our DC to stimulate type II but not NKT cells by an endogenous ligand raises the question, whether this presentation is due to the specific semi-mature stage of the DC. In our previous work we found already that BM-derived DC present an endogenous ligand on CD1d molecules [2]. Although we did not distinguish between type I and II NKT cells, these data clearly indicated that also LPS-maturation leads to the presentation of an unknown endogenous ligand. Therefore we speculate that a surrogate ligand, specific for type II NKT cells, is presented by DC to polarize these cells in a similar pattern as observed for Th1 and Th2 of MHC II restricted T cells.

Selective ligands for type I and II NKT cells may also point to differential interactions of CD1d-expressing APC populations *in vivo*. Especially CD1d^high^-expressing marginal zone B cells are also likely candidates to capture αGC after i.v. injection and to interact with type I NKT cells [39], [40]. Here we found that the CD1d-restricted CD4^+^ T cells are negatively regulated by B7-H1 when expressed by DC presenting an endogenous ligand but did not observe an effect on type I NKT cells *in vivo* or *in vitro* on the different NKT cell lines. In mice αGC injection leads to type I NKT cell-mediated DC maturation [41], [42]. All together, this indicates that the application of the surrogate antigen αGC has different antigenic and immunogenic properties as compared to physiological endogenous ligands by DC.

Together out data point to a novel role of B7-H1 molecules expressed by tolerogenic DC. Unexpectedly, B7-H1 inhibits selectively type II NKT cells but not type I NKT cells. In the absence of this coinhibitory molecule on tolerogenic DC even more protective cytokines are produced. Understanding the mechanisms of differential DC function and the impact of central coinhibitory molecules like B7-H1 in the context of CNS autoimmunity should help applying tolerogenic DC in targeted therapies. Selective induction of boosted tolerogenic properties of DC by modulating PD-1/B7-H1 interactions and thereby constraining induction and development of potentially pathogenic T cells might depict an attractive approach in cell-based therapy.

## Materials and Methods

### Ethics statement

All animals were handled in strict accordance with good animal practice as defined by the relevant national and/or local animal welfare bodies, and all animal work was approved by the appropriate committee (Regierung von Unterfranken, approval no. 55.2-2531.1-73/07).

### Mice

Wild-type C57BL/6 mice were purchased from Harlan Winkelmann (Borchen, Germany). B7-H1^−/−^ mice were kindly provided by L. Chen (Baltimore, USA), CD1d^−/−^ by L. van Kaer (Vanderbilt University School of Medicine, Nashville, TN), Jα281^−/−^ by M. Taniguchi (Institute of Physical and Chemical Research, Kanagawa, Japan) and 2D2 Tg mice by V. K. Kuchroo (Harvard Medical School, USA). Mice were bred and housed under specific pathogen-free conditions in the animal facilities of the Department of Neurology and the Department of Virology and Immunobiology in Würzburg according to German guidelines for animal care.

### Generation and maturation BM-DC

DC were generated from BM cells derived from C57BL/6 or B7-H1^−/−^ mice as described [43]. Briefly, BM cell were flushed from femur and tibia and cultured with 10% supernatant of a GM-CSF producing cell line and used at day 6–10 of culture. For DC maturation, cultures were pulsed for 4 h with TNF (500 U/ml; PeproTech) together with MOG_35–55_ peptide before intravenous injection into the tail vein of mice.

### T-cell proliferation assay

BM-DC from WT and B7-H1^−/−^ mice were stimulated with TNF and loaded with MOG_35–55_ as described above. MOG_35–55_-specific TCR transgenic CD4^+^ T-cells from 2D2 mice were enriched from the spleen with Easy Step CD4^+^ T cell enrichment Kit (Stemcell) and stained with CFSE (Invitrogen) according to the manufacturer's instructions. 4×10^5^ T cells were co-cultured with 3×10^4^, 1×10^4^ or without DC in 96-well plates. After 4 days the cells were stained with anti-CD4 and CFSE-dilution of the CD4^+^ cells was analysed by FACS.

### Induction of EAE and injections of DC

MOG_35–55_ peptide (EVGWYRSPFSRVVHLYRNGK; synthesized and HPLC purified by R. Volkmer, Charite, Berlin, Germany) was used for active induction of EAE. Age- and sex-matched C57BL/6 or CD1d^−/−^ mice were immunized s.c. with 200 µg MOG_35–55_ emulsified in CFA (Sigma-Aldrich, Steinheim, Germany) that was further enriched with *Mycobacterium tuberculosis* H37RA (5 mg/mL) (Difco, Detroit, MI, USA). In addition, mice were injected i.p. with 400 ng pertussis toxin (List Biological Laboratories, Laboratories, Campbell, CA, USA) at the time of immunization (day 0) and 48 h later. Using this standard immunization protocol, we observed a typical chronic disease course, for which clinical signs of disease were monitored daily and scored based on the following scale (EAE score): 0, no disease; 1, limp tail; 2, hind limp weakness; 3, hind limp paralysis; 4, hind and fore limp paralysis; 5, moribund or death. 2–3×10^6^ stimulated and MOG pulsed DC were injected intravenously repeatedly at days −7, −5 and −3 before EAE induction (day 0).

### Isolation of spleen cells and preparation of CNS mononuclear cells

For isolation of CNS mononuclear cells, mice were perfused through the left cardiac ventricle with cold PBS, brains were dissected and spinal cords were flushed out with cold PBS. CNS material was cut into pieces and mononuclear cells were recovered from the interface of a 30–50% percoll gradient centrifuged for 30 min at 5000 rpm. Cells were washed and resuspended in culture medium for further analysis.

Single cell suspensions of splenocytes were obtained by mashing the spleens of donor mice through a 70 µm strainer and subsequent lysis of red blood cells with ACK buffer (150 mM NH_4_Cl, 10 mM KHCO_3_, 0,1 mM EDTA). Splenocytes were cultured in serum-free HL-1 medium (Lonza) supplemented with Penicillin (100 U/ml, PAA), Streptomycin (100 µg/ml, PAA), L-Glutamin (2 mM, PAA) and β-mercaptoethanol (50 µM, Sigma).

### Flow cytometry

BM-derived DC, NKT-cell cell lines, Splenocytes or CNS derived cells were stained with surface antibodies (anti-B7-H1-PE, anti-CD80-FITC, anti-CD86-FITC, anti-CD40-PE, anti-MHC II-PE, anti-CD3-FITC, anti-NK1.1-PerCP-Cy5.5, purchased from BD Pharmingen; anti-B7-DC-PE, anti-PD-1-PE, anti-CD4-PerCp, anti-CD8-PE, purchased from eBioscience) in the presence of FcγRII/FcγRIII-specific antibody (clone 2.4G2) to block unspecific binding. FACS datawere collected on FACS Calibur cytometer (BD Biosciences) and analyzed using FlowJo software (TreeStar) version 7.2.1.

### ELISA and ELISPOT

For the measurement of cytokine responses by ELISA, 4×10^5^ splenocytes depleted from erythrocytes were cultured *in vitro* in the presence of different concentrations of MOG_35–55_ peptide (0–20 µg/ml) and supernatants were collected after 3 days. Sera were collected 2 h after the third immunization. Samples were analyzed for cytokine content using ELISA kits for IL-4, IL-10, IFN-γ (BD Pharmingen), IL-13 and IL-17 (eBioscience).

For ELISPOT assays, 5×10^4^ CNS cells per well were stimulated with 10 µg/mL MOG_35–55_ peptide and cultured for 24 h in 96-well-plates. ELISPOT assay was performed according to the manufacturer's instructions (for IFN-γ: BD Pharmingen; for IL-17: eBioscience). Spots were counted using a Wild Heerbrugg M3Z dissecting microscope or evaluated by CTL Europe GmbH (Aalen, Germany).

### NKT cell line assay

The following NKT cell lines were used: KT12 [44], VIII24 and XV19 [26], BW58 r/m CD28 [45]. NKT cell lines were cultured in RPMI with L-glutamin (GIBCO) containing 5% FCS, 100 U/ml Penicillin/Streptomycin, 0.1 mM non-essential aminoacids, 1 mM Na-pyruvate and 5 µM β-mercaptoethanol. For the co-culture experiments the cell lines were cultured with TNF-matured DC derived from WT or B7-H1^−/−^ mice in medium containing GM-CSF. 5×10^4^ cells from the cell lines KT12, BW58 r/m CD28 or VIII24 or 1×10^4^ cells from the XV19 cell line in an 96 well plate (round bottom) for 24 hours with the DC. As positive control 10 ng/ml αGC (Alexis Biochemicals) was added to the culture. The supernatant of this culture was testet by ELISA for IL-2 production (BD Pharmingen).

### Statistical analysis

Two-tailed Student's t-test was used to determine the statistical significance of difference. A value of p<0.05 was considered significant. Error bars in figures represent standard deviations.
